# Connexin 40 in atrial fibrillation: pathophysiological roles and regulatory mechanisms

**DOI:** 10.1080/07853890.2025.2578728

**Published:** 2025-10-28

**Authors:** Fei Yan, Wei Zhou, Jiulin Chen, Runze Huang, Zhangrong Chen

**Affiliations:** ^a^ The Key Laboratory of Myocardial Remodeling Research, The Affiliated Hospital of Guizhou Medical University, Guiyang, China; ^b^ Department of Cardiovascular Medicine, People’s Hospital of Qianxinan Prefecture, Qianxinan Prefecture, China; ^c^ Department of Cardiovascular Medicine, The Affiliated Hospital of Guizhou Medical University, Guiyang, China

**Keywords:** Connexin 40, atrial fibrillation, cardiac electrophysiology, intercellular communication, proteasomal degradation

## Abstract

Atrial fibrillation (AF), the most prevalent clinical atrial arrhythmia, arises from complex dysregulation of multicellular signalling pathways and molecular networks. Recent advances in cardiac electrophysiology have identified the atrial-specific gap junction protein Connexin 40 (Cx40) as a pivotal regulator of intercellular communication and cardiac electrical synchronization in AF pathogenesis. Aberrant Cx40 expression significantly disrupts atrial electrical conduction, yet its precise regulatory mechanisms remain incompletely understood. This review systematically examines the multifaceted roles of Cx40 in AF development, particularly focusing on how Cx40 genetic polymorphisms influence cardiac electrophysiology and promote atrial structural remodelling. Furthermore, we elucidate the ubiquitination-mediated protein degradation pathways governing Cx40 stability. These insights advance our understanding of AF pathophysiology and highlight potential therapeutic targets for precision arrhythmia management.

## Background

Atrial Fibrillation (AF) represents the most clinically significant atrial arrhythmia, characterized by abnormal electrical activity in the atria that leads to irregular mechanical contractions. This electrophysiological disturbance results in hemodynamic impairment and thromboembolic events, significantly increasing risks of heart failure and stroke [1,2]. With the accelerating pace of population aging, the prevalence of AF continues to rise globally. Current projections estimate that 60 to 120 million individuals worldwide will be affected by this condition by 2050 [3,4]. Existing research has established that AF pathogenesis involves multifaceted interactions between electrophysiological alterations, structural cardiac remodelling, and inflammatory responses, which collectively form an intricate pathophysiological network [5,6]. However, despite substantial progress in identifying predisposing factors and their clinical correlations, significant challenges persist in translating these findings into effective therapeutic strategies.

Gap junction proteins (Connexins, Cxs), as primary components of intercellular communication channels, mediate direct electrical coupling between cardiomyocytes. Among cardiac connexins, Cx40, Cx43, and Cx45 constitute the major subtypes that form gap junctions (GJs), facilitating rapid electrical signal propagation to maintain normal cardiac rhythm and function [7,8]. Compelling evidence demonstrates that abnormal expression patterns of these connexins, particularly Cx40, disrupt atrial electrical conduction homogeneity [9–11] and correlate strongly with pathological processes including atrial fibrosis and electrical remodelling [12]. Elucidating the functional role and regulatory mechanisms of Cx40 in AF pathogenesis through: (1) Integrated Evidence Synthesis: Systematically consolidating emerging evidence on Cx40’s pathophysiological cascades, focusing on conduction heterogeneity-mediated re-entrant mechanisms (e.g. impaired anisotropic conduction-driven microreentry); (2) Regulatory Network Decoding: Multidimensionally deconstructing the hierarchical regulatory architecture governing Cx40 expression, spanning transcriptional control → post-translational modifications (phosphorylation/ubiquitination) → spatial mislocalization; (3) Therapeutic Target Analysis: Presenting Ubiquilin4 (UBQLN4)-mediated Cx40 degradation *via* the ubiquitin-proteasome system pathway (UPS) as a novel therapeutic strategy against AF, while prioritizing research on Cx40 expression regulation.

## Methodology: literature selection process

A systematic search was conducted across seven databases: PubMed, Embase, Web of Science, Cochrane Library, CNKI, Wanfang, and VIP from inception to April 2024. Search terms combined controlled vocabulary (MeSH terms) and free-text words: (‘Connexin 40’ OR ‘Cx40’ OR ‘GJA5’) AND (‘Atrial Fibrillation’ OR ‘AF’) AND (‘Pathophysiology’ OR ‘Regulatory Mechanism’). Inclusion criteria encompassed: (1) Original studies reporting Cx40 alterations in human/animal AF models; (2) Investigations of molecular regulation (e.g. ubiquitination, miRNA); (3) Articles in English or Chinese. Exclusion criteria: Reviews, conference abstracts, studies without control groups, or non-Cx40 focused research. The selection process involved three phases: Title/abstract screening (excluded 61.3% records), full-text evaluation applying inclusion criteria (32.8% retained), and final quality assessment using modified Newcastle-Ottawa Scale (scoring ≥6 required for inclusion). Discrepancies were resolved by consensus with a third investigator.

## Findings

### Basic biological characteristics of Cx40

#### Structure and function of Cx40

**Figure 1.**
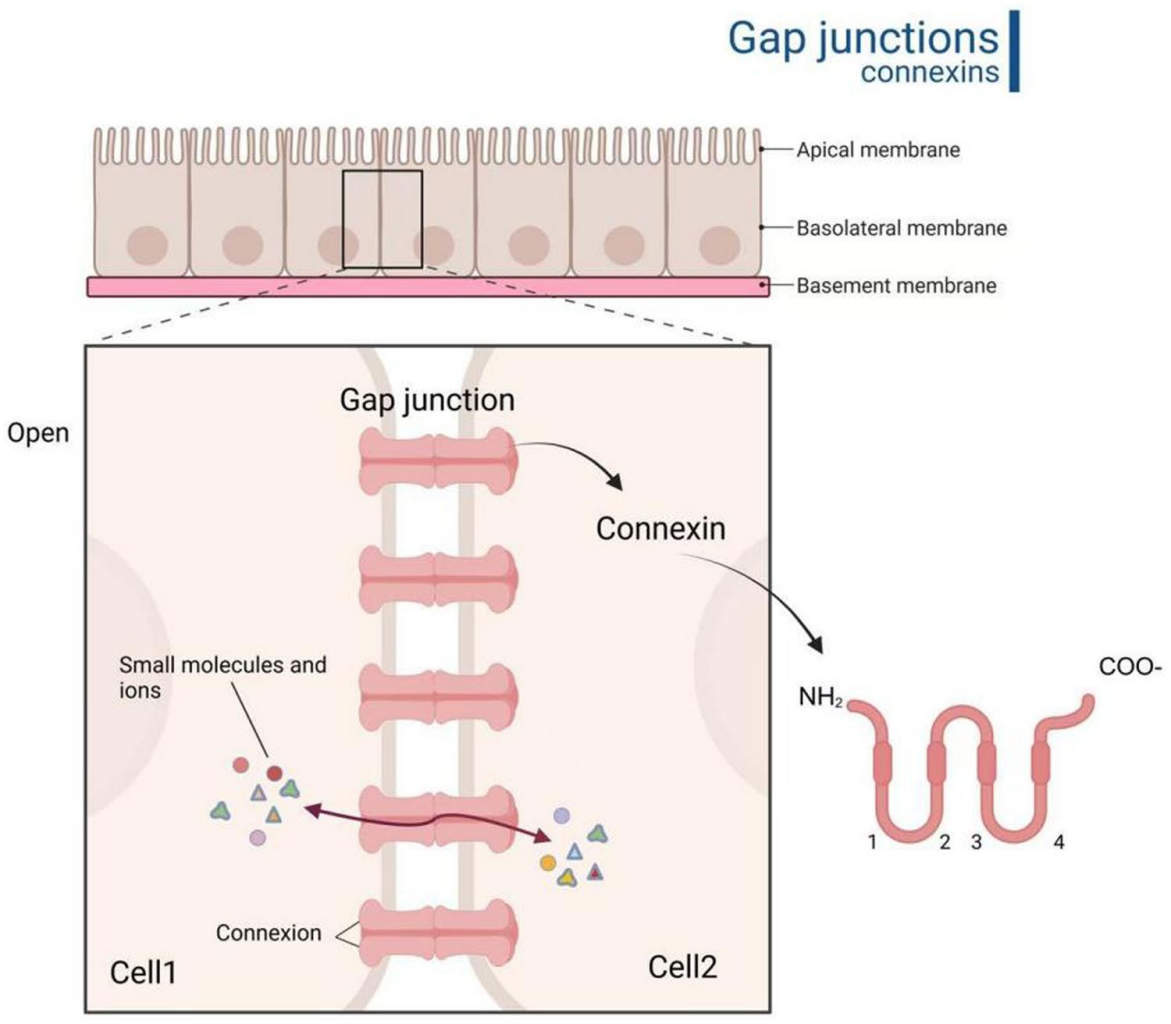
Schematic illustration of connexin hexameric assembly and gap junction ultrastructure. Gap Junction: Intercellular channels formed by docking of plasma membranes from adjacent cells, permitting exchange of small molecules and ions. Connexon: Hemichannel complex consisting of six connexin subunits. Channel patency is established when in the open conformation. Connexin:Tetratransmembrane protein regulating channel permeability through gating mechanisms. Created in BioRender. Yan [18]https://BioRender.com/hseenzr.

Cx40, with a molecular weight of ∼40 kDa, is a crucial gap junction protein belonging to the Cx family. Its structure comprises four highly conserved transmembrane domains, two extracellular loops (with both transmembrane regions and extracellular loops being highly conserved), and intracellular loops and carboxyl termini that exhibit significant variability among different Cx family members. These variable regions are common sites for post-translational modifications, such as phosphorylation and ubiquitination [7]. Following synthesis in the endoplasmic reticulum, Cx40 undergoes modification and packaging in the Golgi apparatus before being transported to the cell membrane. At the membrane, it can assemble independently or synergistically with other Cx family members into hexamers, forming hemichannels. Two adjacent hemichannels dock to create complete gap junctions with a central hydrophobic pore, enabling direct intercellular exchange of small molecules (e.g. ions, metabolites, second messengers) and electrical signal transmission [8,13,14] (Figure 1). Studies reveal that connexin assembly is regulated by multiple factors, including intracellular calcium concentration, pH, and specific signalling pathways [15]. Furthermore, the oligomerization state and channel formation are closely associated with cellular physiological conditions. For instance, under stress or pathological states, altered connexin expression or assembly may impair GJ function, disrupting intercellular communication and tissue homeostasis [16]. Cx40 dynamically regulates atrial electrical synchronization through conformational changes and exhibits specific expression patterns in atrial myocytes and the cardiac conduction system [17].

#### Expression and distribution of Cx40 in the heart

The physiological role of Cx40 in the heart primarily stems from its specialized distribution and involvement in mediating intercellular electrical communication. In cardiac tissue, Cx40 exhibits distinct region-specific expression patterns, predominantly localized at intercalated discs between atrial myocytes and in the conduction system (e.g. sinoatrial node, atrioventricular node). Through end-to-end connections, it enhances longitudinal conduction velocity while restricting transverse conduction, facilitating the orderly propagation of myocardial action potentials to ensure temporal coordination of cardiac contractions [11]. This spatially restricted distribution enables rapid electrical signal propagation *via* low-resistance gap junctions, ensuring precision in cardiac electromechanical coupling [19]. Under pathological conditions, aberrant Cx40 expression strongly correlates with arrhythmogenesis [20]. Specifically, atrial fibrillation patients exhibit disrupted sarcolemmal polarity distribution of Cx40 [21–23]. Such heterogeneous distribution disrupts intercellular electrical coupling, promotes conduction velocity dispersion, and creates a proarrhythmic microenvironment conducive to re-entrant arrhythmias [24].

### Cx40 and atrial fibrillation

#### Association between Cx40 mutations and atrial fibrillation susceptibility

Cx40 is encoded by the GJA5 gene located at chromosome 1q21.2 [25], and its genetic variations exhibit significant correlations with AF susceptibility. Cx40 mutations primarily alter cardiac electrical conduction through impaired electrical coupling. Specifically, these mutations may reduce Cx40 expression, disrupt function, and induce gap junction remodelling, thereby compromising intercellular electrical signal transmission, diminishing coupling efficiency, increasing atrial electrophysiological instability, and triggering cardiac electrical abnormalities [26], ultimately elevating AF susceptibility [27]. In 2006, Gollob and colleagues identified four novel heterozygous missense GJA5 mutations (G38D, P88S, A96S, and M163V) in 15 patients with idiopathic AF [28]. Notably, Shin WH et al. demonstrated that the A96S mutation in mouse models reduced atrial conduction velocity by 43% (0.58 ± 0.07 *vs.* 1.02 ± 0.11 m/s) and prolonged AF duration [29]. The population-specific distribution of Cx40 mutations underscores their importance in AF genetic predisposition. Genome-wide association studies revealed that single nucleotide polymorphisms (SNPs) in the GJA5 locus confer a 2.3-fold increased AF risk in Asian populations [30]. Polymorphisms at positions −44 and −26 of Cx40 are significantly associated with AF occurrence in Asians. Additionally, the interaction between Cx40 and the sodium channel gene SCN5A represents a critical regulatory mechanism for cardiac electrical activity. Studies indicate that Cx40 expression levels are closely correlated with SCN5A expression, and Cx40 mutations may downregulate SCN5A, thereby affecting myocardial depolarization and repolarization [11].

In summary, Cx40 mutations not only impair its normal function but also heighten AF risk by altering cardiac electrophysiological properties. Detection of Cx40 genetic mutations could aid in identifying high-risk individuals and guiding early interventions.

#### Role of Cx40 in atrial fibrillation electrophysiology

The atria predominantly express Cx43 and Cx40. Notably, studies on Cx43 haploinsufficiency or conditional Cx43 knockout mouse models have shown no significant changes in P-wave duration or atrial conduction velocity [31], suggesting that Cx40 serves as the primary connexin mediating electrical signal transmission in atrial myocytes. Under pathological conditions, abnormal expression or functional impairment of Cx40 induces delayed cardiac electrical signalling, promoting electrophysiological abnormalities and increasing susceptibility to AF and other arrhythmias [30,32]. First, the loss or dysfunction of Cx40 significantly reduces intercellular electrical coupling capacity, slowing cardiac conduction velocity, which underscores Cx40’s critical role in myocardial electrical coupling. Second, as a major component of cardiac gap junctions, Cx40 ensures coordinated atrial contraction and efficient pumping by facilitating electrical communication between atrial myocytes. When Cx40 expression is suppressed or its distribution becomes abnormal due to disease, the atrial effective refractory period (ERP) shortens, and action potential propagation duration prolongs. This results in heterogeneous local conduction velocities, uneven electrical coupling, and diffuse conduction, fostering conditions conducive to conduction block and re-entry formation [33]—mechanisms central to AF pathogenesis. These alterations highlight Cx40’s pivotal role in atrial electrophysiology, where its expression and functional integrity directly modulate atrial electrical activity and elevate AF risk [21]. Thus, Cx40 is essential for maintaining normal action potential characteristics and stabilizing atrial electrophysiological homeostasis.

#### Role of Cx40 in atrial remodelling

Atrial remodelling, a core pathological mechanism in AF progression, involves a structural-functional remodelling process characterized by impaired electrical coupling between atrial myocytes and interstitial fibrosis. Cx40 plays a pivotal role in this process. Studies demonstrate that in pathological conditions, such as hypertension [34–36] or diabetes [11,37,38], Cx40 expression is significantly reduced. Cx40 deficiency directly causes dysregulation of the renin-angiotensin system. In Cx40 knockout (Cx40^−/−^) mice, juxtaglomerular cells lack Cx40-mediated gap junctional communication, leading to impaired pressure-dependent renin secretion. This manifests as increased plasma renin activity (+230%) and sustained hypertension (systolic blood pressure elevation ≥25 mmHg) [39,40]. In type 2 diabetic coronary microvasculature, HuR-mediated decrease in Cx40 mRNA stability reduces Cx40 protein expression (immunoblotting shows >70% reduction), resulting in impaired microvascular vasodilation (acetylcholine-induced vasodilatory response reduced by 50%) [37]. Moreover, Cx40 deficiency disrupts endothelial-smooth muscle cell electrical coupling, exacerbating insulin resistance-associated vascular endothelial dysfunction [38]. Simultaneously, clinical evidence discloses a direct correlation between Cx40 expression levels and atrial fibrosis severity [41]. Angiotensin II (Ang II) substantially downregulates atrial Cx40 expression (Western blot detects >50% reduction in Cx40 protein) by activating NF-κB and TGF-β1 pathways while upregulating collagen I and matrix metalloproteinases, directly promoting myocardial fibrosis [41]. C-type natriuretic peptide (CNP) reverses Ang II effects through the AMPK-pPKG pathway, restoring Cx40 expression to baseline levels (+120%) while suppressing TGF-β1 signalling (65% protein reduction) to mitigate fibrosis [42]. This molecular alteration disrupts gap junction integrity, increasing electrical conduction heterogeneity, while simultaneously activating profibrotic signalling pathways. These changes create a vicious cycle where electrical remodelling and structural remodelling mutually reinforce each other—a phenomenon termed ‘AF begets AF’ [43]. Cx40-mediated intercellular coupling disturbances exacerbate conduction velocity dispersion, thereby providing a pathological substrate for AF perpetuation [33]. During atrial remodelling, aberrant expression or lateralized distribution of Cx40 further reduces electrical coupling between atrial myocytes. This impairs action potential propagation, alters atrial electrophysiological properties, and elevates AF susceptibility [20].

#### Altered Cx40 expression in atrial fibrillation patients

Recent studies have highlighted dynamic yet inconsistent changes in Cx40 expression during atrial fibrillation, with bidirectional expression patterns closely linked to AF progression. Multicentre analyses of atrial tissue from AF patients revealed significantly elevated Cx40 levels (up to 2-fold) in those with lone AF or post-cardiac surgery AF compared to sinus rhythm controls, while Cx43 expression remained largely unaffected [44–46]. Chronic AF cohort studies further corroborated this upregulation trend, notably demonstrating higher Cx40 expression in dialysis patients with AF (1.23 ± 0.12) *versus* non-AF dialysis controls (0.74 ± 0.03) and healthy subjects (0.69 ± 0.03, *p* < 0.0001) [47].

However, conflicting data predominantly associate Cx40 downregulation with AF pathogenesis. For instance, Mao and Zhai et al. reported reduced Cx40 expression in chronic AF patients [48], while Dbouk and Mroue et al. observed a 26% decrease in Cx40 levels in AF cohorts *versus* controls [49]. Animal models by Gong and Nakagawa confirmed that Cx40 deficiency prolongs P-wave, PQ/PR intervals, QTc, and QRS duration, increasing susceptibility to atrial tachyarrhythmias [48]. Mechanistic studies by Meşe, Richard, and Rafaqat et al. implicated AMPK activity inhibition and PI3K-AKT-mTOR pathway dysregulation in Cx40 expression disturbances mediated by AGEs and ibrutinib, respectively [21,33].

Despite interstudy variability, these findings collectively indicate that Cx40 dysregulation is intrinsically tied to AF pathogenesis, serving not only as a dynamic biomarker but also as a promising therapeutic target for precision modulation.

#### Cx40 in inflammatory response and oxidative stress

Recent studies have increasingly recognized the roles of inflammation and oxidative stress in atrial fibrillation pathogenesis. Elevated inflammatory biomarkers in AF patients [50,51] not only activate cardiac fibroblasts to promote collagen deposition and atrial fibrosis—key pathological substrates for AF—but also modulate Cx40 expression and function, thereby influencing atrial electrophysiology [11]. For instance, studies in diabetic models have shown that increased inflammatory mediators significantly reduce Cx40 expression, impairing atrial myocyte electrical coupling and exacerbating fibrotic remodeling.Cx40 expression in cardiomyocytes is regulated by oxidative stress, likely through activation of NADPH oxidase (NOX) and pro-inflammatory signalling pathways, such as NF-κB, which amplify cellular responses to oxidative stress [32,52]. Conversely, enhanced intercellular communication *via* Cx40 may upregulate antioxidant enzymes, mitigating oxidative damage [53].

**Figure 2.**
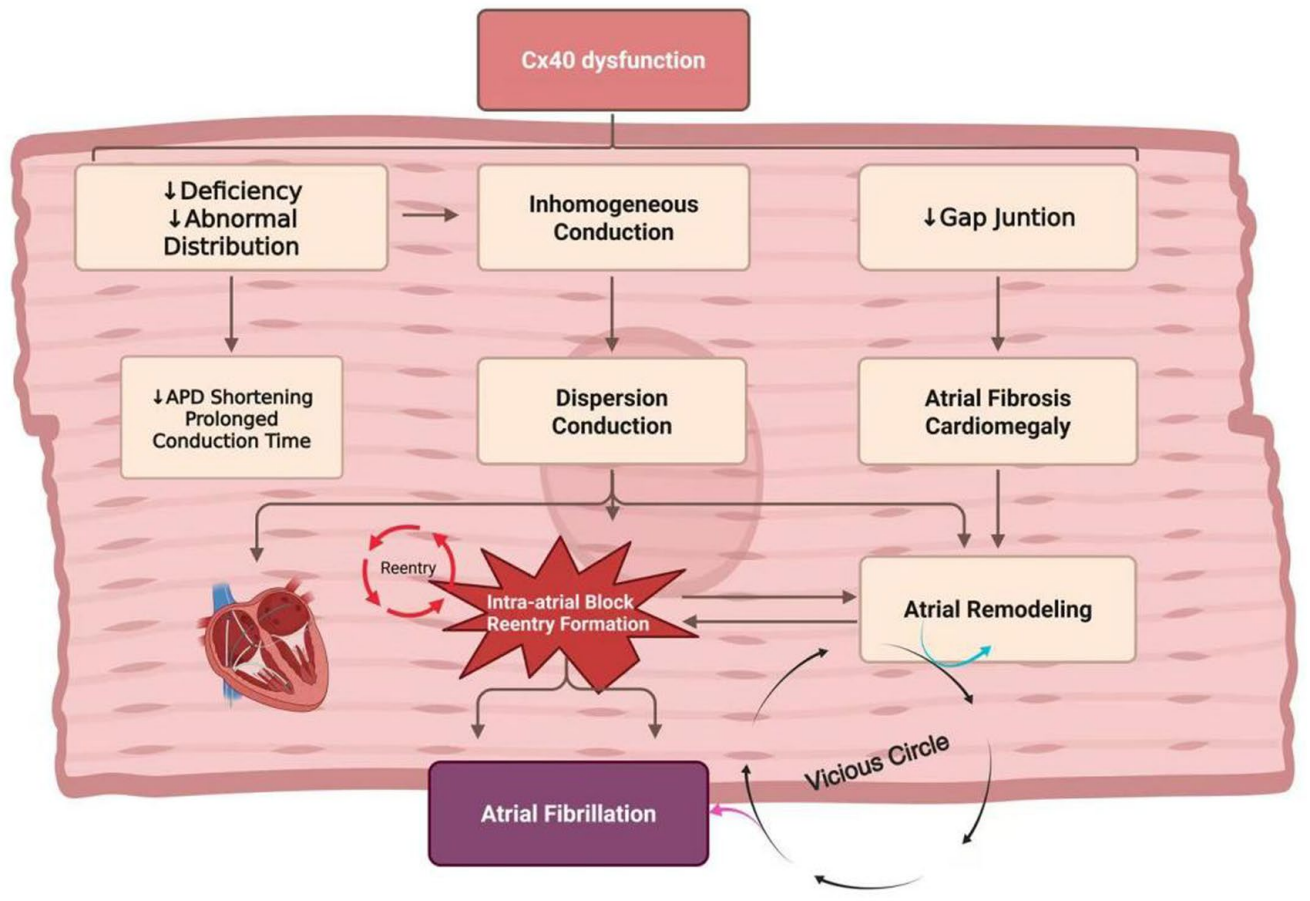
The mechanism of atrial fibrillation pathogenesis associated with Cx40 dysfunction. Functional Cx40 deficiency → Disproportionate connexon clustering → Conduction delay/fragmentation → Focal APD dispersion & fibrotic remodeling → Re-entry *via* unidirectional block → Self-sustained AF circuitry. Created in BioRender. Yan (2025) https://BioRender.com/9m4t4py.

Thus, Cx40 not only governs atrial electrophysiological function but also modulates AF susceptibility through inflammatory and oxidative stress pathways. The association between Cx40 expression variability, functional dysregulation, and AF episodes underscores its potential as a therapeutic target. Strategies to modulate Cx40 expression or function—such as regulating protein degradation—may offer novel interventions for AF. These mechanisms collectively establish Cx40 as a critical regulator and therapeutic target in AF progression (Figure 2).

### Regulatory mechanisms of Cx40

#### Transcriptional regulation of Cx40

The transcriptional regulatory network of Cx40 orchestrates cardiac physiological and pathological processes through synergistic interactions of multiple factors. The core mechanism involves competitive binding of transcription factors, such as Shox2, Foxp3, and KLF4 to the GJA5 gene promoter. A finely balanced antagonism exists between the transcriptional activation by Nkx2-5 and the inhibitory effects of Shox2, which exhibit spatiotemporal specificity during cardiac conduction system development [19,26,54]. Under pathological conditions, TCF21 indirectly modulates Cx40 expression by mediating phenotypic switching in smooth muscle cells—a mechanism prominently activated during atherosclerotic plaque formation. While existing studies confirm these transcription factors regulate Cx40’s critical roles in cardiac development and vascular diseases, remaining gaps persist in understanding their molecular pathways through the Cx40 network in atrial fibrillation pathogenesis, particularly regarding the interplay between energy metabolism remodelling and electrical activity abnormalities.

#### Post-translational modifications and proteasomal degradation of Cx40

Post-translational modifications of Cx40 critically regulate its functional activity and expression levels. For instance, modifications, such as glycosylation and phosphorylation modulate Cx40 stability and function by altering protein charge and spatial conformation, thereby influencing its interactions and activity [55]. Studies demonstrate that Cx40 phosphorylation status is tightly linked to its role in cardiac electrophysiology. Under hyponatremic conditions, altered phosphorylation levels correlate with reduced electrical coupling capacity, while significant phosphorylation changes occur during pathological states like AF. Additionally, oxidative stress and inflammatory factors regulate Cx40 expression by modifying its post-translational modifications, which in turn affect intercellular communication. For example, IFN-γ upregulates Cx40 expression and modulates its function *via* the JAK/STAT signalling pathway, a mechanism vital for endothelial cell homeostasis [56].

**Figure 3.**
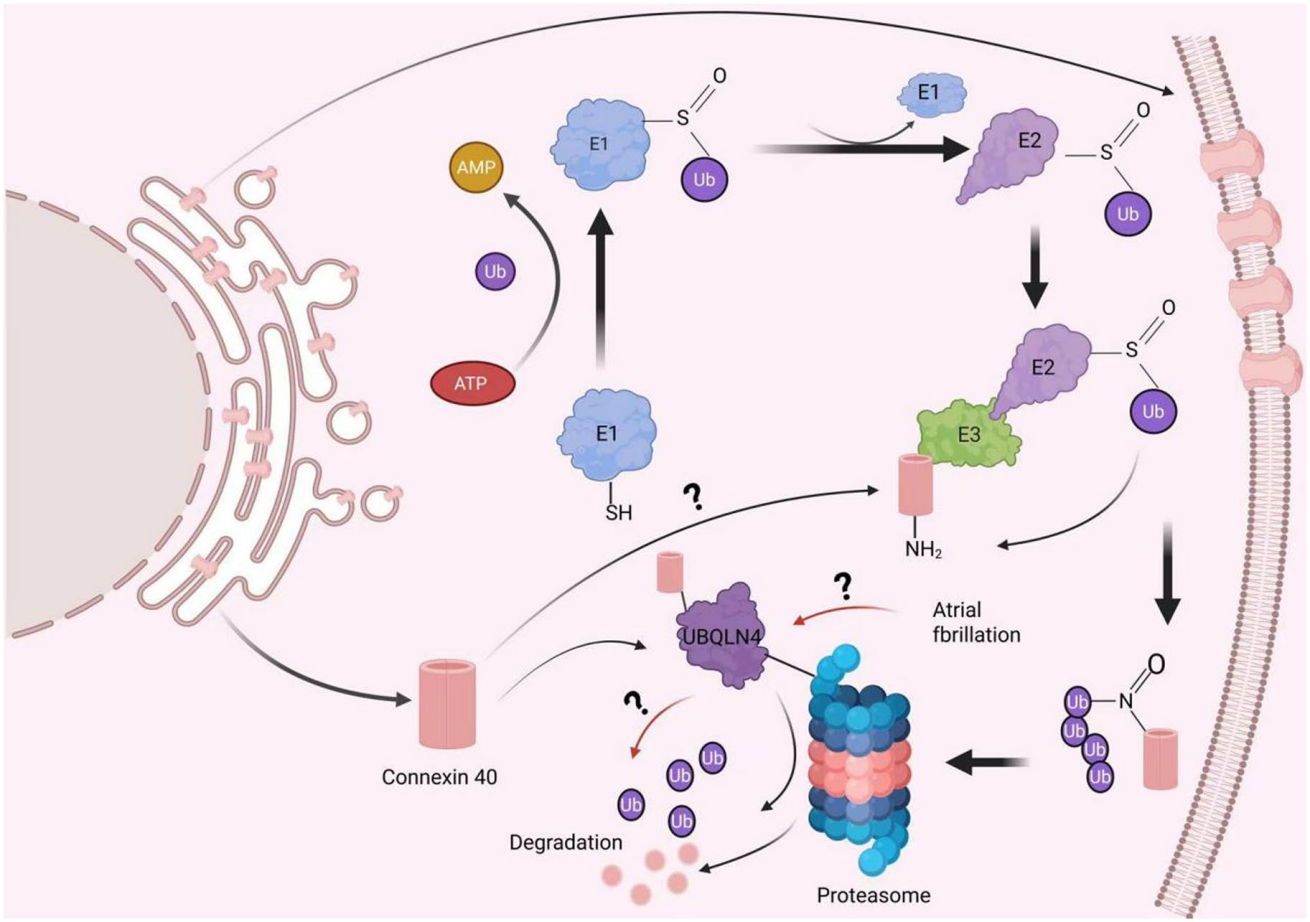
Schematic diagram illustrating the proteasomal degradation of Cx40. UBQLN4 delivers ubiquitinated Cx40 to the proteasome for degradation, potentially impairing atrial gap junction coupling and contributing to AF pathogenesis. Created in BioRender. Yan (2025) https://BioRender.com/de7m2pi.

Degradation of connexins involves dual regulation by endoplasmic reticulum-associated degradation (ERAD) and the UPS. ERAD employs the UPS to selectively recognize misfolded/unfolded proteins, catalyzing substrate ubiquitination *via* an E1-E2-E3 enzymatic cascade (with E3 ligases acting as rate-limiting factors) to form K48-linked polyubiquitin chains [57,58], which are then targeted to proteasomes for degradation. Although the specific E3 ligase and detailed degradation mechanisms for Cx40 remain poorly characterized, recent studies reveal that ubiquitinated Cx40 interacts with the ubiquitin-associated (UBA) domain of UBQLN4. UBQLN4 functions as an adaptor protein, utilizing its ubiquitin-like (UBL) domain to bind the 19S proteasome subunit, thereby delivering ubiquitinated Cx40 for proteasomal degradation [59]. Notably, the molecular dynamics of this pathway under AF conditions—including E3 ligase recognition elements and stress-induced post-translational modifications—remain unresolved (Figure 3).

### Clinical application prospects of Cx40

Studies demonstrate that Cx40 plays a pivotal role in the pathogenesis and progression of atrial fibrillation. Its expression levels, spatial distribution, and functional status are closely associated with atrial structural remodelling and electrophysiological abnormalities, collectively contributing to AF pathology. Emerging Cx40-targeted therapeutic strategies include:

1. Mechanistic studies showing ROCK contributes to cardiorenal remodeling by inhibiting MYPT-1 phosphorylation, thereby increasing myosin light-chain kinase activity [60]. Left atrial appendage biopsies from AF patients *versus* those in sinus rhythm confirm ROCK pathway involvement in AF pathogenesis [61]. Further studies demonstrate enhanced MYPT-1 phosphorylation (a ROCK activity marker) and increased Cx40 expression in AF patients [47]. ROCK inhibitor fasudil treatment reduces phosphorylated MYPT-1 levels, subsequently decreasing Cx40 protein expression and improving cardiac function; in-depth exploration of the ROCK pathway in AF may elucidate arrhythmogenic mechanisms, positioning Rho kinase inhibitors as potential therapeutic targets [47];
2. Connexin downregulation associating with inflammatory cytokines, such as interleukin-6 (IL-6) and tumor necrosis factor-α (TNF-α) [62], which promote cardiac remodeling and fibrosis ultimately leading to arrhythmias. SGLT2 inhibition may improve adipose tissue function and modulate serum leptin, adiponectin, and IL-6 levels, thereby beneficially influencing cardiovascular risk [63]. Accordingly, preliminary studies indicate attenuated Cx downregulation in mice following SGLT2 inhibitor intervention [64], potentially mediated by modified inflammatory cytokines post-therapy; consequently, SGLT2 inhibitors may regulate Cx40-mediated electrophysiological properties and structural remodeling, exhibiting antiarrhythmic effects [64];
3. Gene therapy using tools like the rAAV9-Gja5 vector to enhance sinoatrial node-specific Cx40 expression, thereby ameliorating arrhythmias [65].

These findings lay a theoretical foundation for developing novel Cx40-targeted therapies, potentially offering new avenues for AF prevention and treatment. Furthermore, the biomarker potential of Cx40 in AF patients is gaining increasing recognition. Studies indicate that reduced Cx40 expression correlates not only with AF occurrence but also with AF duration and recurrence risk [12], suggesting Cx40 quantification may facilitate early identification of high-risk AF patients [66]. Additionally, Cx40 detection can be integrated with other biomarkers, such as atrial troponin and N-terminal pro-brain natriuretic peptide (NT-proBNP) to establish comprehensive evaluation models, thereby enhancing AF predictive capability [11]. This multi-marker panel strategy could provide clinicians with more precise AF risk assessment tools, supporting personalized treatment regimens and positioning Cx40 as an emerging critical biomarker in AF management. However, current clinical evidence remains limited, necessitating large-scale randomized controlled trials to validate Cx40’s clinical utility.

It is critical to emphasize that although recent studies have highlighted the significant role of Cx40 in atrial fibrillation pathogenesis, its expression levels exhibit marked heterogeneity across different investigations, potentially linked to interspecies variations, underlying comorbidities in study populations, and model discrepancies. However, the regulatory mechanisms underlying these expression differences and their specific contributions to AF development remain poorly defined. Furthermore, systematic insights into how dynamic synthesis-degradation regulation of Cx40 participates in AF pathophysiology are lacking.

Future research should prioritize elucidating Cx40’s precise mechanisms in diverse pathological contexts by standardizing study cohorts, optimizing homogenized animal models, and dissecting its upstream regulatory networks to clarify clinical relevance. Concurrently, deciphering synergistic interactions and comparative advantages between Cx40 and other molecular markers will advance our understanding of AF’s complex pathology and enhance clinical predictive accuracy. Large-scale multicentre studies are warranted to systematically evaluate the efficacy and safety of Cx40-targeted therapeutic strategies.

## Conclusion and prospects

It is critical to emphasize that although recent studies have highlighted the significant role of Cx40 in AF pathogenesis, academia remains divided on whether Cx40 abnormalities constitute a cause or consequence of AF. Cx40 plays a critical role in electrical signal transmission among atrial cardiomyocytes; its reduced expression diminishes intercellular electrical coupling capacity, consequently elevating AF risk [27,65]. Specific Cx40 gene mutations may alter protein function, increasing AF susceptibility [49]. Conversely, AF onset may reciprocally influence Cx40 expression. Multiple studies examining post-AF Cx40 alterations demonstrate an inverse correlation between Cx40 levels and atrial remodelling severity in AF patients, suggesting that persistent AF contributes to Cx40 expression abnormalities [67], thereby establishing a complex bidirectional relationship. Future investigations should integrate gene-editing tools with population-scale transcriptomic screening to longitudinally track Cx40 trajectories from pre-AF stages through arrhythmia onset in de novo cases, thereby clarifying its etiological role. Concomitantly, its expression levels exhibit significant heterogeneity across different studies. For instance, significantly elevated Cx40 levels have been observed in patients with lone atrial fibrillation, post-cardiac surgery atrial fibrillation, and chronic dialysis-associated atrial fibrillation [35–38], whereas other clinical investigations and experimental AF models (animal/cellular) predominantly demonstrate reduced Cx40 levels [21,33,39]. Owing to the inherent limitations of animal and cellular models in replicating complex comorbidities, this expression heterogeneity in AF is postulated to stem from interspecies variation, underlying comorbidities within study populations, and methodological differences across experimental paradigms. However, the regulatory mechanisms underlying these expression differences and their specific contributions to AF development remain poorly defined. Furthermore, systematic insights into how dynamic synthesis-degradation regulation of Cx40 participates in AF pathophysiology are lacking. Future research should prioritize elucidating Cx40’s precise mechanisms in diverse pathological contexts by standardizing study cohorts, optimizing homogenized animal models, and dissecting its upstream regulatory networks to clarify clinical relevance. Concurrently, deciphering synergistic interactions and comparative advantages between Cx40 and other molecular markers will advance our understanding of AF’s complex pathology and enhance clinical predictive accuracy. Large-scale multicentre studies are warranted to systematically evaluate the efficacy and safety of Cx40-targeted therapeutic strategies. Current evidence confirms characteristic Cx40 expression alterations in AF patients, with its correlation to cardiac electrical remodelling underscoring dual potential as both a therapeutic target and prognostic biomarker. Nevertheless, integrating foundational and clinical research remains essential to construct a comprehensive Cx40 regulatory network map, thereby providing theoretical foundations for establishing precision diagnosis and treatment frameworks for AF.

## Limitations

This review acknowledges several constraints: Potential omission of non-English literature and recent preprints may bias interpretations of Cx40 regulation; Definitive evidence distinguishing whether Cx40 abnormality is a cause or consequence of AF remains lacking in current research. UBQLN4’s involvement in AF pathogenesis remains hypothetical without direct *in vivo* human validation; Temporal-spatial dynamics of Cx40 synthesis/degradation in AF-specific microenvironments are uncharacterized; Current models inadequately recapitulate human atrial substrate complexity. Future research priorities include: (1) Future investigations should integrate gene-editing tools with population-scale transcriptomic screening to longitudinally track Cx40 trajectories from pre-AF stages through arrhythmia onset in de novo cases; (2) Validating UBQLN4 mechanisms: Perform multiplexed imaging analysis on atrial fibrillation patient biopsies and establish humanized genetically modified murine models; (3) Deciphering Cx40 regulatory networks: Implement single-cell tracking and proximity labelling techniques (e.g. BioID/APEX) in atrial-specific cell lines or tissue specimens.

## Data Availability

Data sharing is not applicable to this article as no new data were created or analyzed in this study.
